# Clinical Gaze in Risk-Factor Haze: Swedish GPs' Perceptions of Prescribing Cardiovascular Preventive Drugs

**DOI:** 10.1155/2012/612572

**Published:** 2012-11-18

**Authors:** Josabeth Hultberg, Carl Edvard Rudebeck

**Affiliations:** ^1^Åby Health Care Centre, County Council of Östergötland, Department of Medical and Health Sciences, General Practice, Faculty of Health Sciences, Linköping University, 581 85 Linköping, Sweden; ^2^Institute of Community Medicine, University of Tromsoe, Tromsoe, Norway; ^3^Research Unit of Kalmar County Council, Kalmar, Sweden

## Abstract

*Aims*. To explore general practitioners' (GPs') descriptions of their thoughts and action when prescribing cardiovascular preventive drugs. *Methods*. Qualitative content analysis of transcribed group interviews with 14 participants from two primary health care centres in the southeast of Sweden. *Results*. GPs' prescribing of cardiovascular preventive drugs, from their own descriptions, involved “the patient as calculated” and “the inclination to prescribe,” which were negotiated in the interaction with “the patient in front of me.” In situations with high cardiovascular risk, the GPs reported a tendency to adopt a directive consultation style. In situations with low cardiovascular risk and great uncertainty about the net benefit of preventive drugs, the GPs described a preference for an informed patient choice. *Conclusions*. Our findings suggest that GPs mainly involve patients at low and uncertain risk of cardiovascular disease in treatment decisions, whereas patient involvement tends to decrease when GPs judge the cardiovascular risk as high. Our findings may serve as a memento for clinicians, and we suggest them to be considered in training in communication skills.

## 1. Introduction

General practitioners are heavily involved in considering and prescribing cardiovascular preventive drugs and maintaining their increasing use [1]. Numerous trials have shown indisputable effects of cardiovascular preventive drugs [2]. How to apply these findings in clinical medicine remains an issue of controversy. Proponents of increased drug use calculate on reduced cardiovascular mortality whereas sceptics fear a threat to public health and to the sustainability of health care by introducing preventive drug treatment to large proportions of healthy populations [3, 4]. A Swedish survey showed that between 1980 and 2000, the number of people who stated that they had a chronic disease almost doubled. This was parallel to an increase in preventive drug utilisation, inferring that people comprehend the preventive medication as a treatment for disease [5].

There is a movement towards increased patient participation in health-care decisions in practice and in the legislation of many countries including Sweden [6]. Governments, health-care organisations and insurance companies have a common interest in public health and costeffectiveness in prescribing. Guidelines aim to implement evidence-based prescribing [7] and rational drug use [8] but are supported by drug trials largely sponsored by the pharmaceutical industry [9]. 

Demands on GPs' prescribing are thus numerous, multi-faceted, and sometimes contradictory [10, 11]. The collective result of how patients and GPs handle cardiovascular prevention constitutes the basis for the subsequent drug utilisation in the population. Most previous qualitative studies of how GPs prescribe cardiovascular preventive drugs have focused on barriers to a desirable implementation of guidelines [12, 13]. For a broader understanding of cardiovascular preventive drug prescribing, it needs to be investigated with different approaches, and from GPs' own professional perspectives, with no defined preferences regarding their behaviour.

## 2. Aim

We aimed to explore GPs' descriptions of their thoughts and actions when prescribing cardiovascular preventive drugs.

## 3. Methods

### 3.1. Interviews

We chose group interviews with GPs practising together to collect data that would capture individual and collective professional experience. Fourteen GPs from two primary health care centres in south-eastern Sweden participated, see Table 1. One is situated in a suburb near a fairly large city and is the first author's workplace. The other is in the centre of a small city. They serve urban, suburban, and rural populations. The interviews were held at the participants' workplaces, the first in 2004 and led by the second author, the second in 2007 and led by the first author. Audio recordings were transcribed verbatim shortly after each interview.

The topic, thoughts and actions when prescribing cardiovascular preventive drugs, was held in focus with minimal guidance and occasional use of a checklist, Table 2. Quieter participants were encouraged to express their thoughts. The participants expressed conflicting opinions and views with little or no hesitance and the material proved to be rich in content relevant to the research question.

### 3.2. Analysis

Qualitative content analysis was considered appropriate to the aim [14]. The first interview was analysed before the second was planned. After the second interview, the material from both interviews was analysed. The transcripts were thoroughly read and then slightly condensed. Meaning units were identified and assigned codes. Codes were grouped into subcategories and subcategories into main categories, resulting in a stepwise abstraction from the original text. Internal homogeneity and external heterogeneity between categories were sought. At each step, the results were checked against the original material and adjusted according to it, similarly to the model of constant comparison [15]. The results of each analytical step described above were discussed and negotiated between the authors until agreement was reached.  Table 3 exemplifies each level of abstraction. Quotations were selected to illustrate the findings.

### 3.3. Ethics

Oral and written information to participants stated that their participation was voluntary and that they were free to withdraw at any time without explanation. The original audio recordings and transcripts have been handled by the authors only. In all presentations of this study, quotations have been slightly altered when necessary to retain participants' anonymity.

## 4. Results

### 4.1. The Patient as Calculated

#### 4.1.1. Measurability

When describing their assessments of patients, the GPs considered a number of risk factors for cardiovascular disease, and different aspects of them. Among the qualities of the risk factors discussed, measurability was crucial for decision making on prescribing. Separate threshold values for blood pressure and cholesterol or estimates of total risk of future disease, based on more comprehensive risk estimations, were used when deciding on prescribing.

*“These are treatments ruled by guidelines with firm levels for decision, 140/80 and diabetes, that's crystal clear.”*

Also those who regarded a comprehensive risk calculation as the proper basis for decision making reported referring to thresholds in guidelines when motivating decisions to patients. There were elaborate descriptions of followup of prescriptions with specific reference to measurable factors.

#### 4.1.2. Solidity of Knowledge

It was recognised that there is an inherent uncertainty in probabilistic reasoning and application of epidemiological data to individuals.

* “I'm waiting for the day, and probably won't experience it during my career, when we are able to define who is really at risk.”*

Lack of agreement and consensus about preventive treatment within the profession and the scientific community was acknowledged.

*“This issue of cholesterol—there are different opinions about it.”*

Not-readily-measurable factors such as stressful life events and other psychosocial factors were considered but were felt to be more difficult to base prescribing decisions on. Various degrees of personal limitation were perceived regarding knowledge, access to relevant information in the prescribing situation, and ability to convey such information to patients.

*“I'd like to have the risk figures in front of me, not just at the back of my head.”*

### 4.2. Inclination to Prescribe

Each doctor entered the prescribing situation with a set of attitudes that taken together contributed to a general inclination to prescribe drugs. This observation rests on statements about cardiovascular prevention and comparisons with other drugs and nonpharmacological interventions.

#### 4.2.1. Attitudes towards Prevention

There was consensus concerning the priority of symptom-relieving drugs before preventive drugs and of secondary prevention before primary prevention. Yet attitudes towards preventive medicine diverged. One argument for reluctance to engage in preventive medicine was the risk of harming healthy people when labelling them with diagnoses. Another was lack of resources in primary care, yet another that taking drugs instead of changing an inappropriate habit was an easy way out and no cure. These arguments all implied low inclination to prescribe.

*“They (occupational health services and private practices) measure cholesterol values in healthy ladies and young guys and refer them to us …doesn't make you very happy, does it? It's irresponsible.”*

Reasons for engagement in preventive medicine were not only to do good, that is, to prevent disease, but also to do what is *right* according to guidelines. Emphasis on these seemingly similar arguments differed.

*“I put it like this: people that know more than I have assessed this and it's something we give to everyone in this situation to diminish risks….”*

*“I have something else. I try to involve the patient in the choice …I think it increases the will to take it and if I think it is good I also want the patient to understand why.”*

#### 4.2.2. Attitudes towards Drugs

Pharmacological treatments were regarded as powerful. A strict indication and avoidance of unnecessary prescriptions were judged important. Side effects were spontaneously and thoroughly discussed in the interviews.

*“It's really dangerous when people come to hospitals or nursing homes and all of a sudden the nurses start giving them everything they've been prescribed ….”*

The participants' own experience of drug use influenced the inclination to prescribe

*“…after I had my disease …my attitude towards drugs is less negative.”*

Lifestyle change (change in diet, physical exercise, smoking, and alcohol intake) was regarded either as an alternative or a supplement to medication. As an alternative, it was seen as preferable, sparing the patient from drug treatment, or at least postponing it. Some described lifestyle change as harmless, and a possible cure of risk conditions; opinions that indicate low inclination to prescribe but not necessarily reluctance to engage in preventive medicine.

*“You don't start medication till you've made several measurements, and if you think you can lower cholesterol with lifestyle change you do that first.”*

### 4.3. The Patient in front of Me

#### 4.3.1. A Personal Relation

A personal relation and personal encounters with patients were described as a must for the prescribing of new drugs. The participants often referred to the patient as “the person in front of me” when describing clinical situations. Sending prescriptions by mail was dismissed as exceptions made only when there was an established relation or when a followup appointment was scheduled. Mutual exchange of information was viewed as fundamental for prescribing decisions.

*“It depends on the patient in front of me, their personality, my experience of them, and whether I have met them before.”*

#### 4.3.2. Deciding Who Decides

The GPs' descriptions of their behaviour when prescribing suggested different views on the responsibility for decision making. Adjustments of decision allocation between patient and doctor depended on the individual patient and situations arising in the clinical encounter.

The view of the decision as the doctor's responsibility was motivated by the doctor's better knowledge and duty to achieve the best possible adherence to, and result of, treatment.

*“He's feeling quite well actually, but he still takes some of the drugs …in my opinion he absolutely has to continue with them because he still has his damaged vessels ….”*

Some participants stated that they saw the patient as the ultimate decision maker, and that the doctor's responsibility was limited to conveying correct and necessary information for the patient's decision.

* “I am not in charge in this case, but an advisor. I'm someone the patient wishes to get advice from …and I must not force anything on the patient.”*

Consultations with decision making on treatment were depicted as negotiations. Some described reaching agreement as a goal per se. Agreement, it was also argued, increases patient satisfaction and adherence to prescribed treatments. In choices between equal options according to the doctor, or when treatment was considered advantageous but not essential, the patient might be given more influence on the decision. Correct information to patients about their options was emphasized. 

When the patient's risk was estimated by the doctor to be high or when patient adherence to treatment was felt to be low, the GPs described adjustments towards a directive consultation style.

* “I might think that it's important also for that person to value his life, but there I might take charge and take on a more patriarchal role because it's important to keep the coronary arteries in shape as long as possible and avoid an infarction.”*

Fear of making patients worried was one reason for not involving them. Uncertain or lacking facts on risk combined with an experienced need to reassure the patient sometimes led to increased decisiveness, and the adoption of a directive style. Assessments of patients' capacity to comprehend necessary information and of their wish to participate also affected the doctor's decision on who should decide about treatment.

*“How do you introduce that way of seeing it with the patient in a complicated reality, when the patient has several diseases and many drugs, half of which at least are treatments for some hazy risks?”*

## 5. Discussion

### 5.1. Comments on Findings

We present the ingredients of cardiovascular preventive drug prescribing as seen from the GP perspective. The aim, methods, and size of the study did not allow a description of individual GPs styles nor interpretations on gender or age specific attitudes. Consultation behaviour was described to fall along a continuum from an authoritative physician's decision to an informed patient's decision. With “shared decision making” as an intermediate, this conforms to the model of consultation styles described by Charles et al. [16]. Similarly, Silwer et al. found GPs to have different views on the allocation of responsibility (between doctors and patients) when decisions on primary cardiovascular prevention were to be made [17]. However, the GPs in our study described situational adjustments to their prescribing behaviour. Agreement, a shared decision, and flexibility when prescribing may be regarded as goals in themselves and may also lead to better outcome in terms of perceived health and patient satisfaction [18–20]. Flexibility had limitations. Situations with perceived high cardiovascular risk or low drug adherence were in our study reported as leading to a directive consultation style. With regard to the benefits of shared decision making, such adjustments may be counterproductive.

In the shared decision making model, the doctor is to identify equipoise between treatment alternatives before sharing the decision with the patient. This places the interpretive prerogative about equipoise with the doctor and thus also the power to decide [21, 22]. Our results confirm this reasoning among the GPs in the process of “deciding who decides”. In situations with low cardiovascular risk, as judged by the GP, he or she tended to decide not to decide, or not to take full responsibility for decision making. As a consequence, patients were more likely to be invited to partake in decision making when less was at stake but the choice was more delicate and laden with uncertainty. Exceptions were when the GP perceived the patient to be unwilling, incapable, or too anxious to decide. These situational adjustments of decision making are understandable, but from the patient's point of view, neither fully rational nor desirable. As with the possibly counterproductive adjustments when cardiovascular risk is high, they cannot be solved by modifications of guidelines or the implementation of them, which has been suggested [17]. 

Our aim was to explore GPs' drug prescribing. Nonpharmacological treatments were brought up and discussed in relation to drug treatments. GPs' choice between lifestyle interventions and drug prescribing has been reported as a dilemma and a possible obstacle to drug treatment when lifestyle change is regarded as preferable [13]. Such reasoning requires that lifestyle change and drugs are regarded as conflicting and mutually exclusive choices. Our results confirm this as one, but not the only, way of seeing it. Parallel initiation of lifestyle change and drug therapy was also reported. This is in accordance with Silwer et al. who found that GPs regarded pharmaceutical and nonpharmaceutical prevention as independent options [17]. 

Clinical inertia, defined as “clinicians' failure to initiate or intensify drug treatment when indicated”, has been put forward as an obstacle to adequate prescribing, and to treatment targets being reached in practice [23, 24]. The inherent uncertainty of probabilistic reasoning has been suggested as one rationale for clinical inertia [10, 11, 13]. “Soft” reasons have also been referred to [23]. Our GPs expressed concern about the lack of non-measurable facts in risk algorithms, evoking patients' anxiety with talk of risk, harming frail elderly people with drugs, causing side effects in asymptomatic people, and labelling them with risk diagnoses. Such “soft” arguments against drug treatment emanating from GPs' interaction with “the patient in front of them” may be well as solid as the alleged “hard” facts of “the patient as calculated” [25, 26]. 

The decision to prescribe has been described as a product of clinical interaction [27]. Our results were in accordance with this. Ultimately the relation with “the patient in front of me” appeared to be the frame of the decision on prescribing and the weight of numeric values of “the patient as calculated” were subjected to negotiation. In fact, discourse-analytical studies of audio recordings from clinical consultations have shown treatment decisions to be based on negotiated blood-pressure values rather than actual measured values [28].

### 5.2. Comments on Methods

We interviewed groups of colleagues working together. In group interviews controversies may be suppressed and a consensual understanding reinforced [13]. On the other hand, in-house hierarchy and consensual understanding are the basis for clinical action [29]. It is purposeful to collect data in peer groups that reflects and articulates the norms that guide their practice. There were disagreements and descriptions of shortcomings, indicating an open discussion climate and that the participants did not experience the interviews as a knowledge test. The latter has been proposed as a risk when interviewing peers [30]. 

The material proved to be sufficiently rich and varied to suffice for the purpose. Further data collection would possibly have added detail to the result, but we find it unlikely that the major features of our findings would have changed. 

For practical reasons, the interval between the interviews was three years. The specific contents of the categories, such as informants' views about treatment thresholds, may change with alterations of guidelines and other influences. We regard it as a strength that all categories were derived from both group interviews and thus proved to be stable over the period.

This interview study renders information about GPs' thoughts and views on prescribing, but only their described behaviour. Further studies of real life consultations are needed to explore how GPs and patients handle decision making on cardiovascular preventive drugs. How do the ethical dilemmas of public health epidemiology come to expression in clinical practice where individuals interact?

## 6. Conclusion

GPs' prescribing of cardiovascular preventive drugs, from their own descriptions, involved “the patient as calculated” and “the inclination to prescribe,” which were negotiated in the interaction with “the patient in front of me.”

In situations with high cardiovascular risk, the GPs described a tendency to adopt a directive consultation style to make the patient adhere to treatment. In situations with low cardiovascular risk, the GPs reported inclination to retreat from the shared decision making model to take on a mainly informative role. Adjustments towards directive consultation styles, regardless of the motive, may decrease patient involvement and have unintended counterproductive results. By adjustments towards informed patient choice in low risk situations, the patients became relatively more responsible when there was great uncertainty on the benefits of preventive drugs. We conclude that these situational adjustments in decision making are understandable, but from the patient's point of view, neither fully rational nor desirable. With reservation for the degree of transferability of our findings, they may yet serve as a memento for clinicians, and we suggest them to be considered in the training of communication skills. Our findings imply patterns of how the responsibility for decision making is shared between doctors and patients. Further studies of this with regard to drug prescribing should be undertaken in actual clinical interaction.

## Figures and Tables

**Table 1:** Characteristics of participants.

Gender	Years of work experience
Primary health care centre 1	

Male	26
Female	27
Male	11
Male	26
Female	24
Male	5
Male	1

Primary health care centre 2	

Male	22
Male	12
Male	39
Female	30
Female	32
Female	11
Male	34

**Table 2:** Interview checklist.

Opening questions	
How do you do when you prescribe a new medication for continuous use?	
Can you recall a recent case when you prescribed medication to prevent cardiovascular disease?	

Areas for further probing	
Descriptions of routine, rules of thumb, phrasing, information about drugs.	
Specifics of preventive treatment: the concept of risk, life long treatment.	
Consideration of patients' responses, attitudes, adherence.	

**Table 3:** Example of the derivation of a main category.

Meaning unit	*“I am waiting for the day, and probably won't experience it during my career, when we are able to define who is really at risk.” *
Condensed meaning unit	Cardiovascular risk for the individual patient is unknown
Code	Inherent uncertainty
Subcategory	Solidity of knowledge
Main category	The patient as calculated
